# Supplementary material for: Shigella sonnei infection of zebrafish reveals that O-antigen mediates neutrophil tolerance and dysentery incidence
Source: PLoS Pathog. 2019 Dec 12;15(12):e1008006. doi: 10.1371/journal.ppat.1008006 (PMC6980646; doi:10.1371/journal.ppat.1008006)

Figure S1. *S. sonnei* is more virulent than *S. flexneri* in a zebrafish infection model

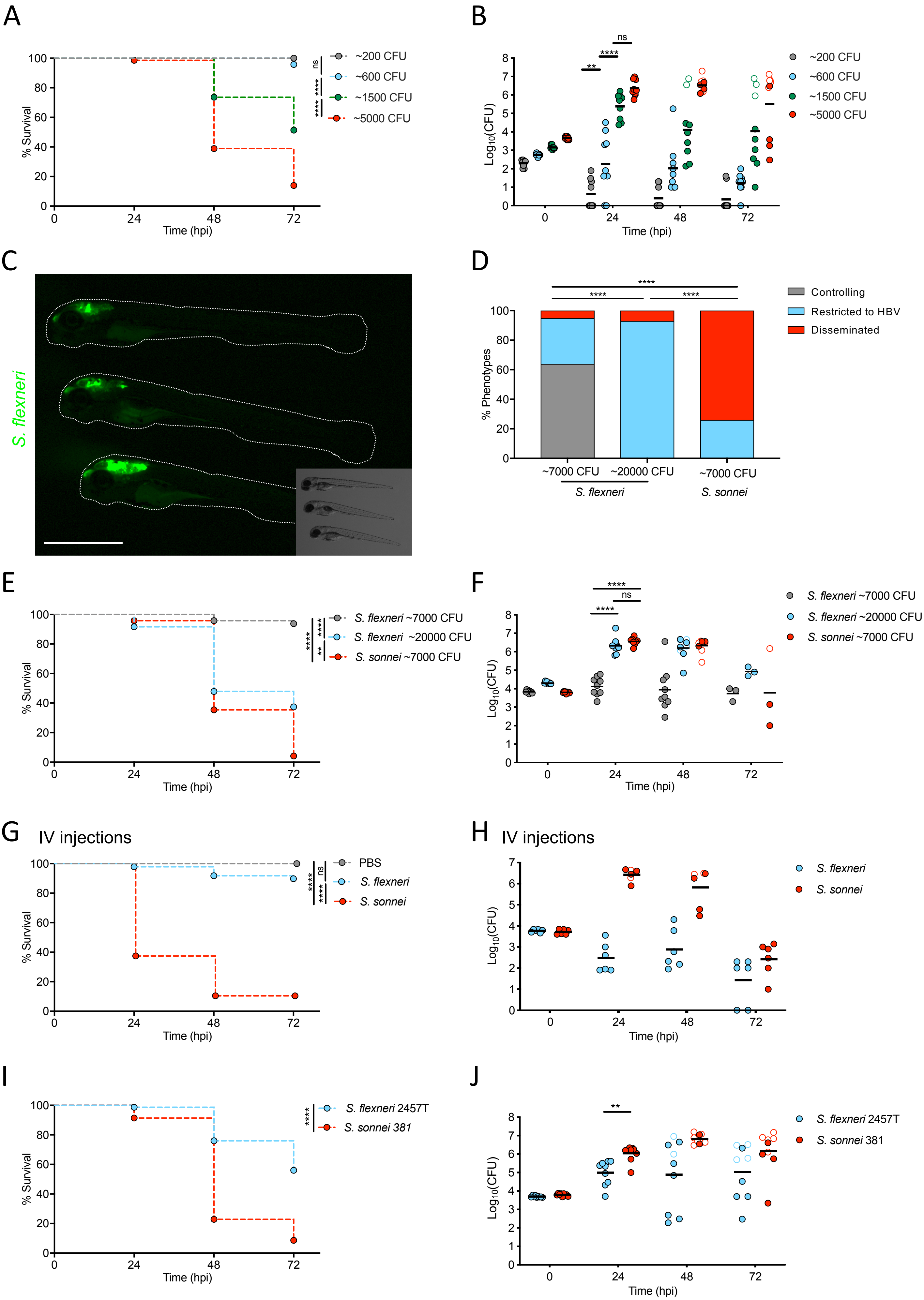

Figure S1 (continued). *S. sonnei* is more virulent than *S. flexneri* in a zebrafish infection model

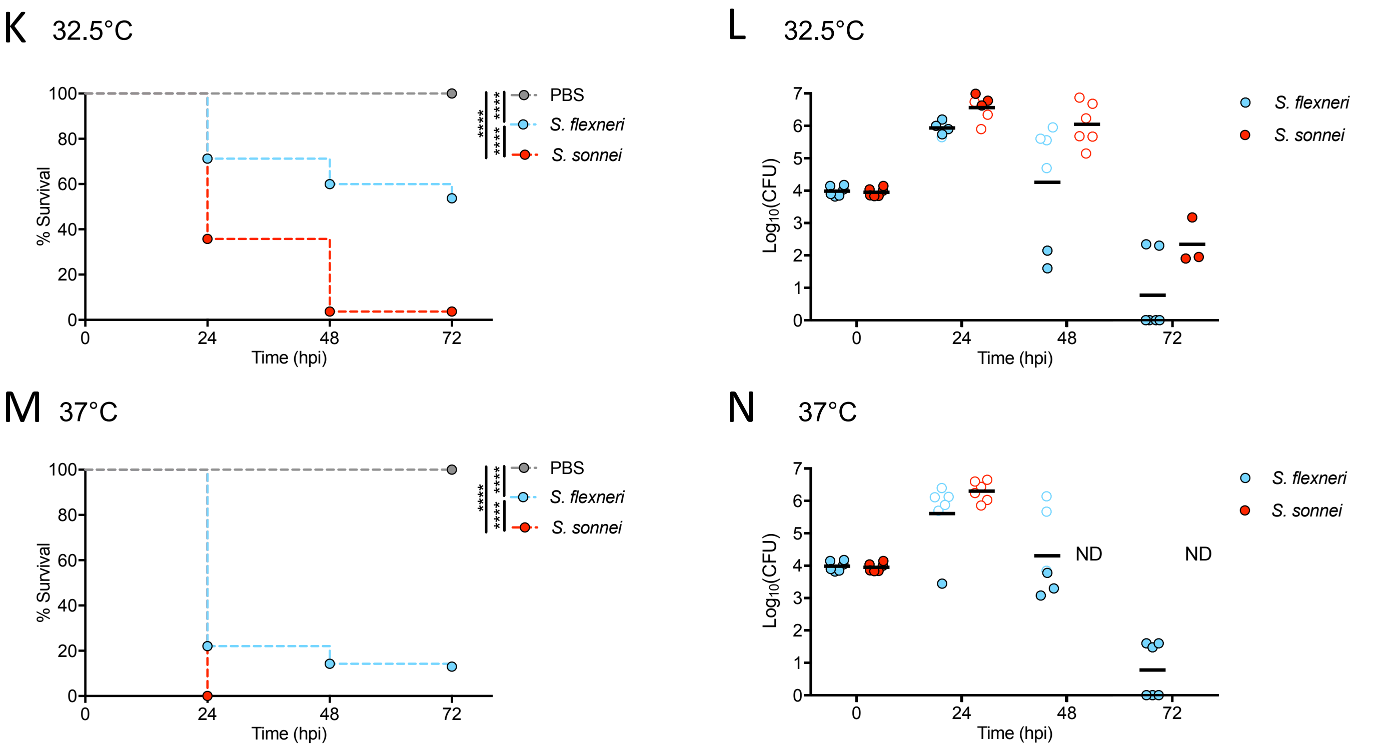

Supplement: S1 Fig — A,B. Dose response to S. sonnei infection. Survival curves (A) and Log10-transformed CFU counts (B) of larvae injected in the HBV with increasing doses of S. sonnei. ~200 CFU range: 100–300 CFU (grey); ~600 CFU range: 400–700 CFU (blue); ~1500 CFU range: 1000–2000 CFU (green); ~5000 CFU: 4000–6000 CFU (red). Experiments are cumulative of 3 biological replicates. In B, full symbols represent live larvae and empty symbols represent larvae that at the plating timepoint had died within the last 16 hours. Statistics: Log-rank (Mantel-Cox) test (A); one-way ANOVA with Sidak’s correction on Log10-transformed data (B); ns, non-significant; **p<0.0021; ****p<0.0001. C-F. S. sonnei can disseminate from the injection site. Representative images of three larvae injected in the HBV with ~20000 CFU of GFP-labelled S. flexneri (C, compare to Fig 1E and 1F). The frequency of larvae with bacterial dissemination out of the HBV at 24 hpi is significantly higher for the S. sonnei-infected group when compared to the S. flexneri infected group (even when S. flexneri input is ~3-fold higher than S. sonnei input) (D). Survival curves (E) and Log10-transformed CFU counts (F) of larvae injected in the HBV with ~7000 CFU S. flexneri (grey), ~20000 CFU of S. flexneri (blue) or ~7000 CFU S. sonnei (red). Experiments are cumulative of 3 biological replicates. In E, full symbols represent live larvae and empty symbols represent larvae that at the plating timepoint had died within the last 16 hours. Due to poor larval survival at 72 hpi, CFU data are available for < 3 larvae per biological replicate. Statistics: two-sided chi-squared contingency test (D); Log-rank (Mantel-Cox) test (E); one-way ANOVA with Sidak’s correction on Log10-transformed data (F); ns, non-significant; **p<0.0021; ****p<0.0001. Scale bar = 1 mm. G,H. S. sonnei is more virulent than S. flexneri in an intravenous infection model. Survival curves (G) and Log10-transformed CFU counts (H) of larvae injected intravenously (IV, via [file ppat.1008006.s001.pdf]
