# Supplementary material for: Shigella sonnei infection of zebrafish reveals that O-antigen mediates neutrophil tolerance and dysentery incidence
Source: PLoS Pathog. 2019 Dec 12;15(12):e1008006. doi: 10.1371/journal.ppat.1008006 (PMC6980646; doi:10.1371/journal.ppat.1008006)

# Figure S2. Whole animal dual-RNAseq profiling of *S. sonnei* infected larvae

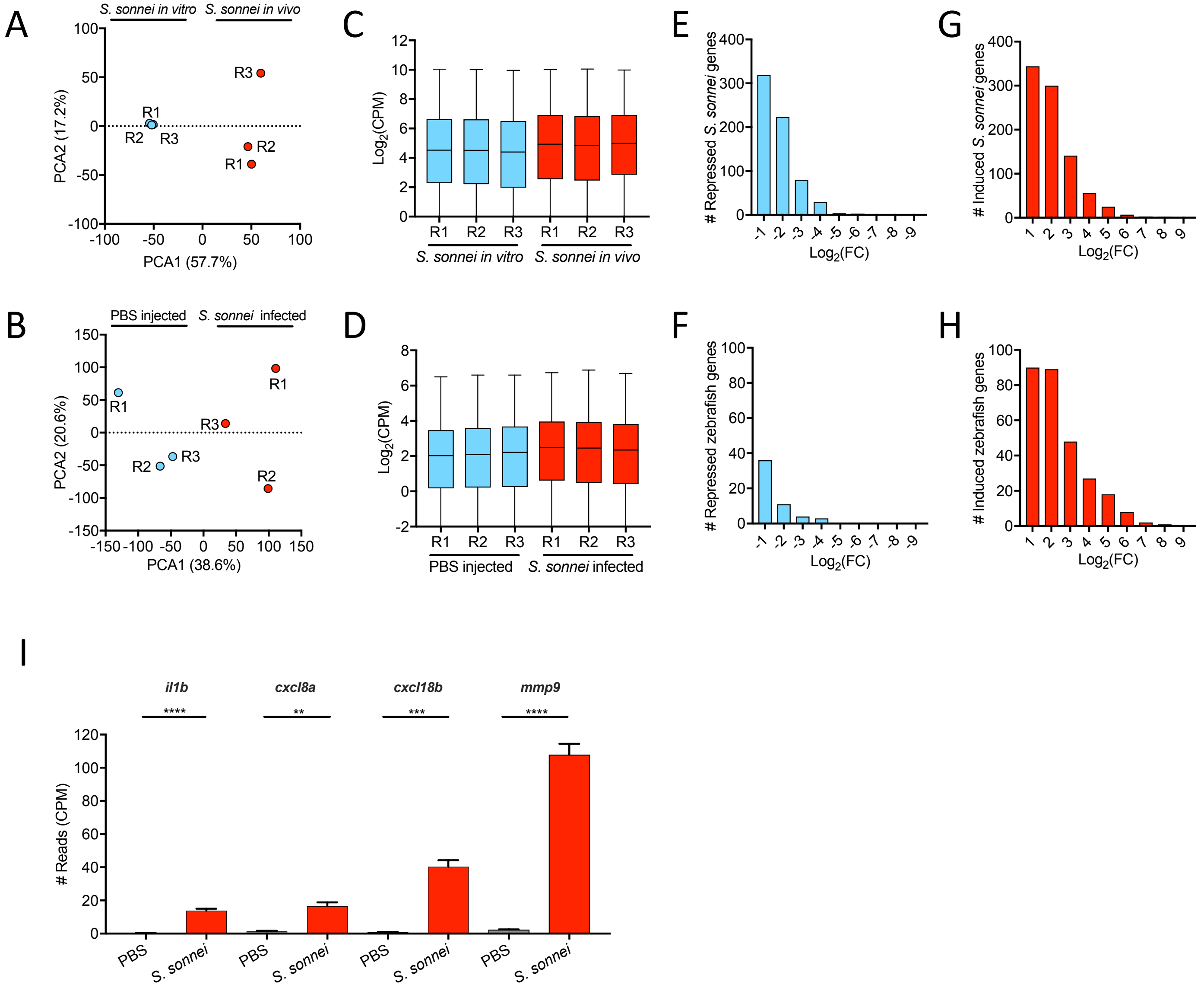

## Figure S2 (continued). Whole animal dual-RNAseq profiling of *S. sonnei* infected larvae

J

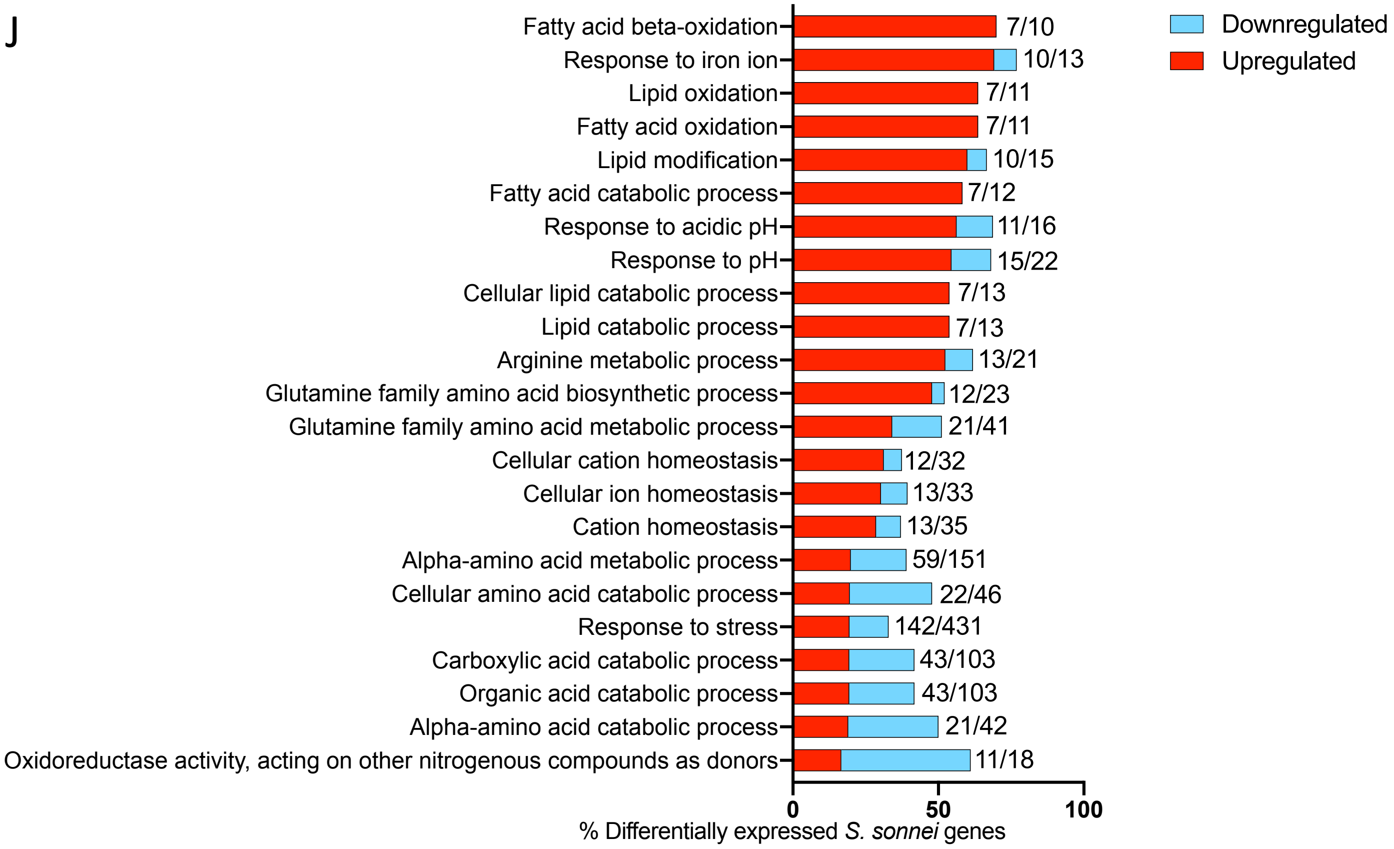


K

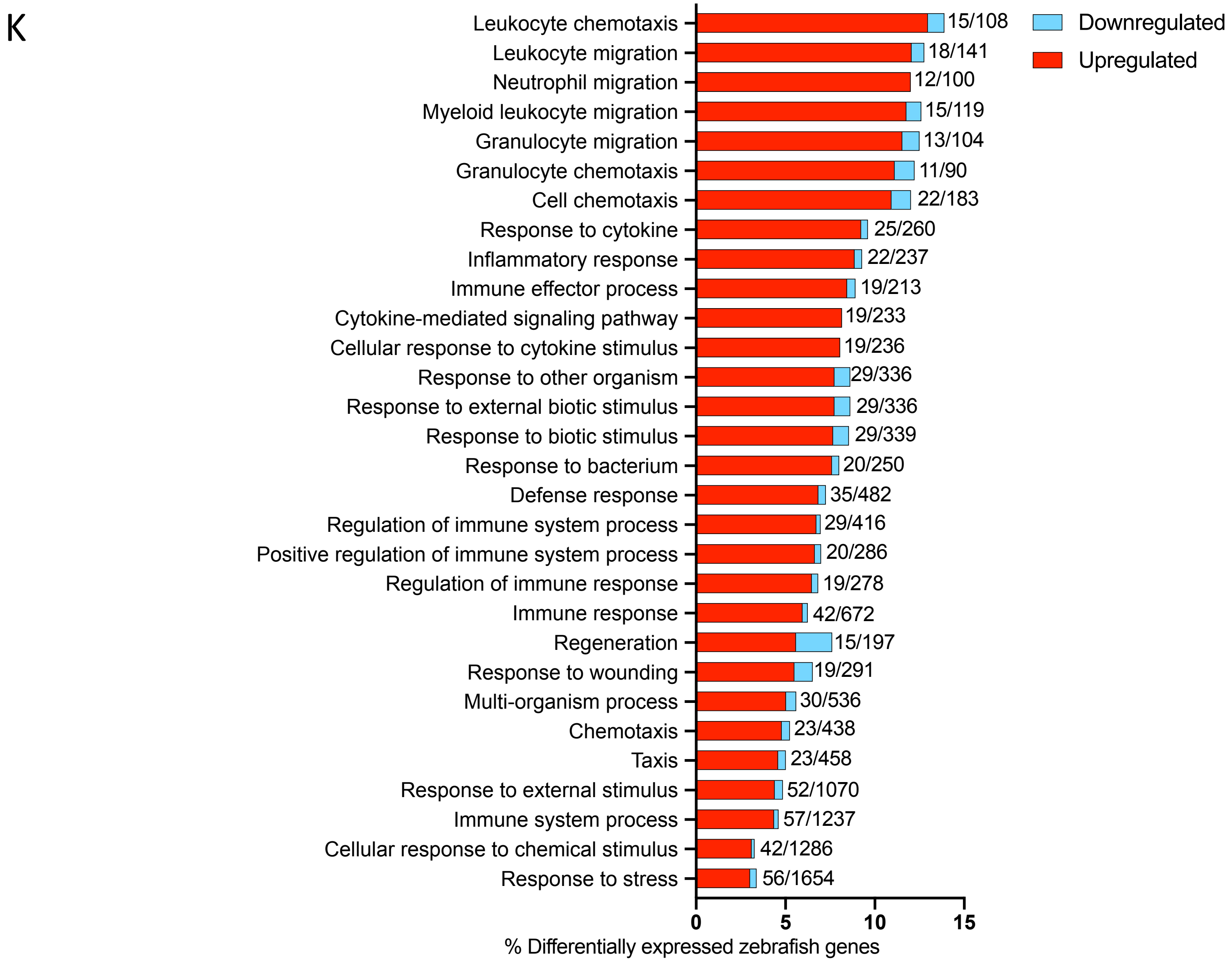

Supplement: S2 Fig — A,B. Principal component analysis (PCA) of S. sonnei and zebrafish larvae transcriptomes. Analysis was performed on counts per million (CPM) reads values, using the dedicated PCA tools in R. Individual biological replicates (R1, R2, R3) for control (blue) and infected (red) conditions are reported. % in brackets indicate the variance of dimension explained by each principal component. Plot in A refers to S. sonnei genes and plot in B refers to zebrafish genes. C,D. Boxplots representing the distribution of reads within the RNAseq libraries of each individual sample. Boxplots represent the sample median CPM reads with interquartile range, while whiskers indicate the 2.5–97.5 percentile range. Control samples are indicated in blue and infection samples are indicted in red. Biological replicates (R1, R2, R3) are also indicated. Plot in C refers to S. sonnei gene libraries and plot in D refers to zebrafish gene libraries. E-H. Distribution histograms of significantly differentially expressed genes in S. sonnei and zebrafish larvae during infections. Each bar represents the number of significantly differentially expressed genes (repressed, blue (E,F); induced, red (G,H)) in each interval of Log2(FC). Plots in E,G refer to S. sonnei genes, while plots in F,H refer to zebrafish genes. I. Induction of well-established inflammatory markers in the RNAseq transcriptome. Bars indicate the average CPM reads for representative inflammatory marker. Compare to induction of same genes tested independently by qRT-PCR at the same timepoint in Fig 1D. Statistics: unpaired t-test on Log2-transformed data; **p<0.0021; ***p<0.0002; ****p< 0.0001. J. Pathway enrichment analysis of S. sonnei during infection in vivo. Pathway enrichment analysis was performed using eggNOG-mapper v2 to infer gene functions based on orthology. % are relative to all genes bioinformatically annotated to the pathway of interest. A variety of stress response processes are induced in S. sonnei in vivo in the zebraf [file ppat.1008006.s002.pdf]
