# Supplementary material for: Shigella sonnei infection of zebrafish reveals that O-antigen mediates neutrophil tolerance and dysentery incidence
Source: PLoS Pathog. 2019 Dec 12;15(12):e1008006. doi: 10.1371/journal.ppat.1008006 (PMC6980646; doi:10.1371/journal.ppat.1008006)

Figure S3. *S. sonnei* virulence depends on its O-antigen

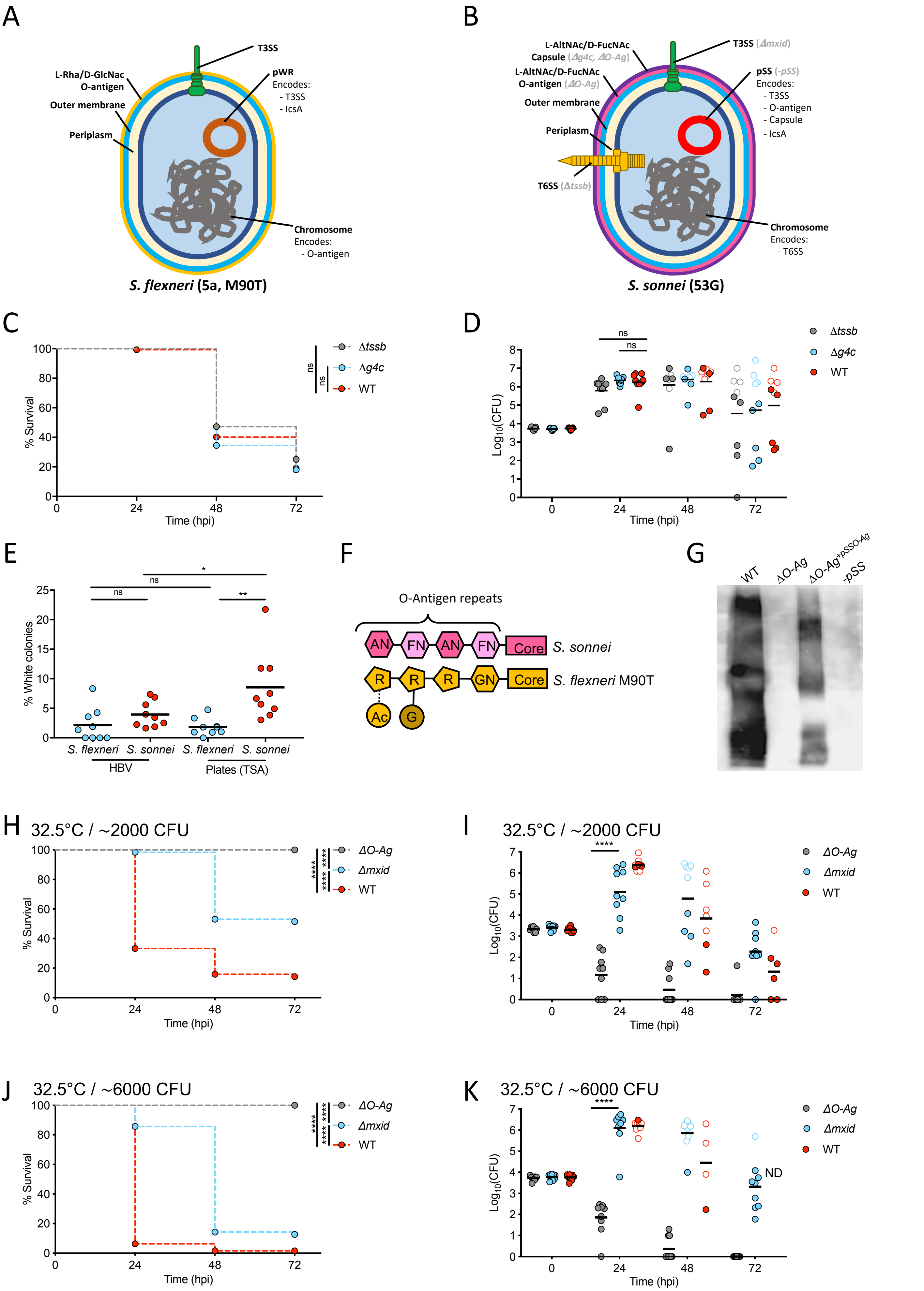

Figure S3 (continued). *S. sonnei* virulence depends on its O-antigen

L

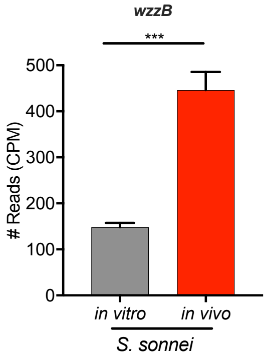

M

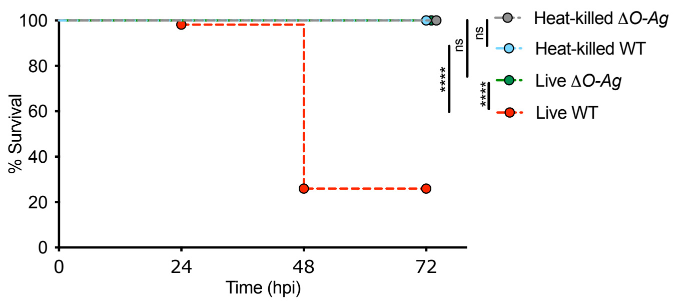

Supplement: S3 Fig — A,B. Schematic of S. sonnei and S. flexneri. Both S. flexneri and S. sonnei virulence plasmid encodes a type 3 secretion system (T3SS). However, differently than S. flexneri, S. sonnei virulence plasmid (pSS) encodes genes for the biosynthesis of a capsule and O-antigen (O-Ag) non-homologous to those of other Escherichia and Shigella species. S. sonnei additionally encodes a type 6 secretion system (T6SS) on the bacterial chromosome. The schematic in B also reports (in grey and between brackets) the name of the mutants used in the study. C,D. Virulence of S. sonnei in zebrafish does not depend on the T6SS or capsule. Survival curves (C) and Log10-transformed CFU counts (D) of larvae injected in the HBV with S. sonnei Δtssb (grey), Δg4c (blue), or WT (red) strains. Experiments are cumulative of 4 (C) or 3 (D) biological replicates. In D, full symbols represent live larvae and empty symbols represent larvae that at the plating timepoint had died within the last 16 hours. Statistics: Log-rank (Mantel-Cox) test (C); one-way ANOVA with Sidak’s correction on Log10-transformed data (D); ns, non-significant. E. S. sonnei virulence plasmid is maintained in vivo in zebrafish at 28.5°C. Larvae were injected with 1 nl (~7000 CFU) in the HBV with S. flexneri (blue) or S. sonnei (red) for 24 h at 28.5°C. Control bacteria (TSA plates) were spotted onto tryptic soy agar plates (10 μl of the bacterial inoculum/spot) and also grown for 24 h at 28.5°C. Bacteria were then harvested from larvae or plates and grown on Congo-Red plates at 37°C to quantify colonies that lost the virulence plasmid. Experiments are cumulative of 2 biological replicates. Statistics: one-way ANOVA with Sidak’s correction; ns, non-significant; *p<0.0332; **p<0.0021. F. Comparison of S. sonnei and S. flexneri O-antigen. S. sonnei 53G O-antigen has a unique sugar composition compared to the O-antigen of S. flexneri M90T. Figure legend abbreviations: AN: 2-Acetamido-2-deoxy-L-altruronic acid (L-AltNAc); FN: 2-Acet [file ppat.1008006.s003.pdf]
