# Supplementary figures and images for: Shigella sonnei infection of zebrafish reveals that O-antigen mediates neutrophil tolerance and dysentery incidence
Source: PLoS Pathog. 2019 Dec 12;15(12):e1008006. doi: 10.1371/journal.ppat.1008006 (PMC6980646; doi:10.1371/journal.ppat.1008006)

**Figure S4. *S. sonnei* O-antigen can counteract clearance by zebrafish neutrophils**

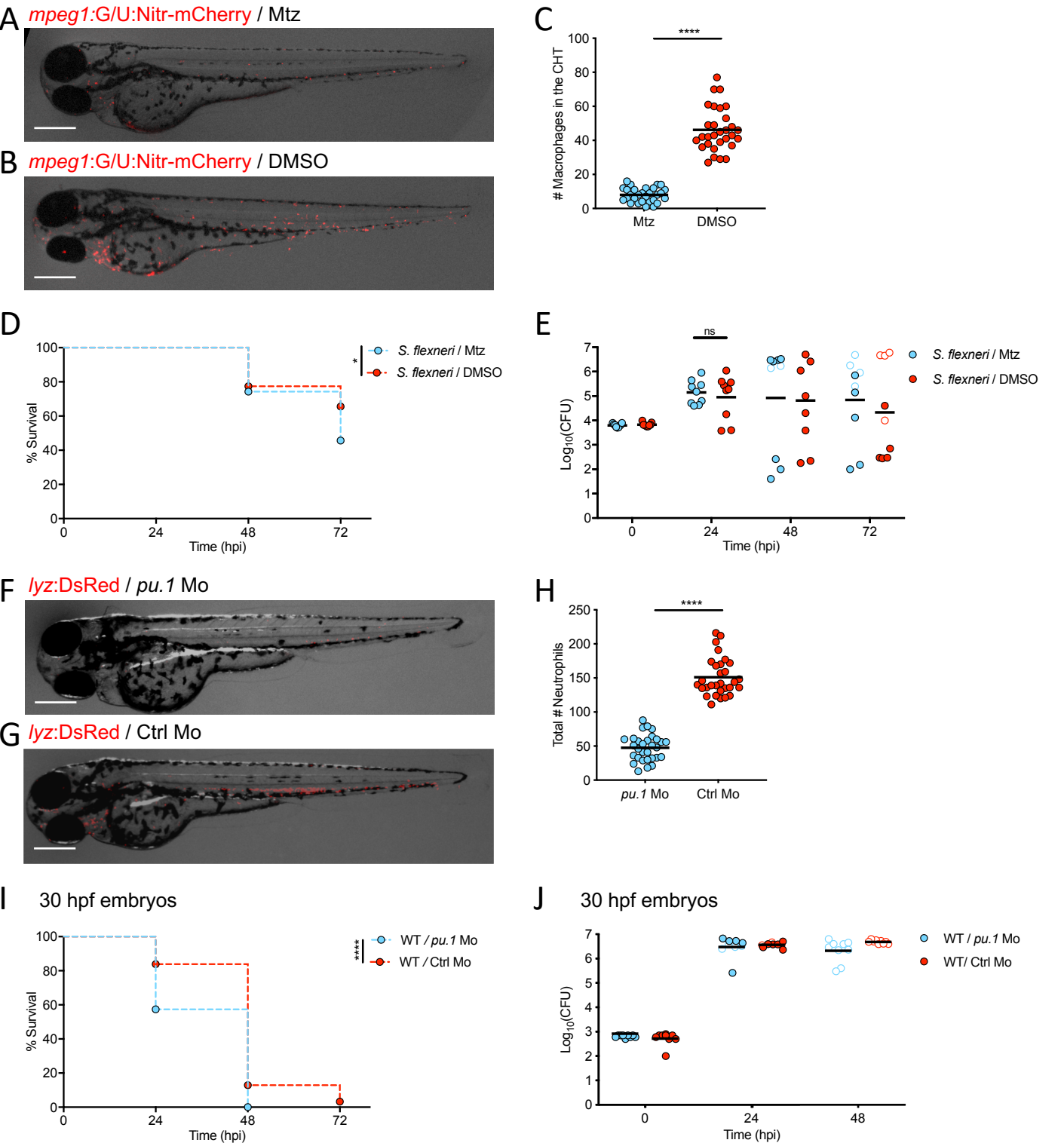

Supplement: S4 Fig — A-C. Chemical ablation of macrophages. Representative images (A,B) and quantification (C) of macrophage ablation in Tg(mpeg1:Gal4-FF)gl25/Tg(UAS-E1b:nfsB.mCherry)c264 larvae which were treated with either Metronidazole (Mtz, macrophage ablated group, blue) or control DMSO vehicle (DMSO, red) prior to infection in the HBV with S. sonnei. Experiments are cumulative of 2 biological replicates. Statistics: two-tailed Mann-Whitney test; ****p<0.0001. Scale bars = 250 μm. D,E. Macrophage ablation increases susceptibility to S. flexneri. Survival curves (D) and Log10-transformed CFU counts (E) of Tg(mpeg1:Gal4-FF)gl25/Tg(UAS-E1b:nfsB.mCherry)c264 larvae which were treated with either Metronidazole (Mtz, macrophage ablated group, blue) or control DMSO vehicle (DMSO, red) prior to infection in the HBV with S. flexneri. Experiments are cumulative of 3 biological replicates. In E, full symbols represent live larvae and empty symbols represent larvae that at the plating timepoint had died within the last 16 hours. Statistics: Log-rank (Mantel-Cox) test (D); unpaired t-test on Log10-transformed data (E); ns, non-significant; *p<0.0332. F-H. pu.1 morpholino knockdown results in neutrophil depletion. Representative images (F,G) and quantification (H) of neutrophil depletion in Tg(lyz:dsRed)nz50 larvae injected with pu.1 morpholino (blue) or control morpholino (red) at 1-cell stage. A significant ~3-fold decrease in neutrophil number can be observed at 3 dpf, prior to infection in the HBV with S. sonnei. Experiments are cumulative of 3 biological replicates. Statistics: two-tailed Mann-Whitney test; ****p<0.0001. Scale bars = 250 μm. I,J. pu.1 morpholino knockdown increases susceptibility to S. sonnei when infections are performed at 30 hpf. Survival curves (I) and Log10-transformed CFU counts (J) of pu.1 morphant (blue) or control (red) larvae infected in the HBV with WT S. sonnei. Experiments are cumulative of 3 biological replicates. In J, full symbols represent live larvae and [file ppat.1008006.s004.pdf]

Figure S6. Innate immunity can be trained to control *S. sonnei* *in vivo*

A

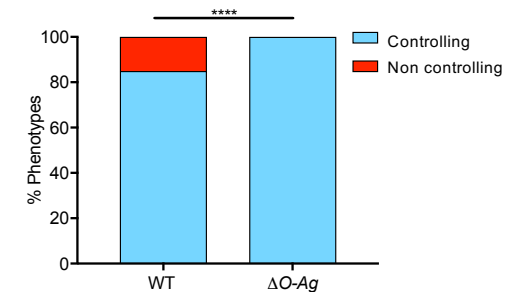

B

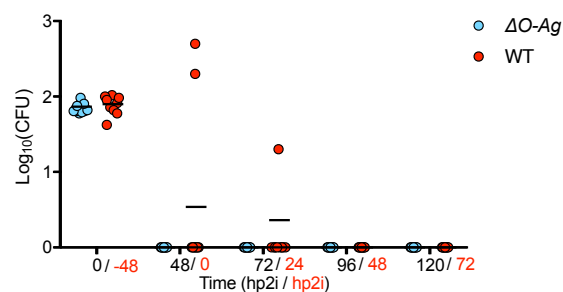

Supplement: S6 Fig — A,B. Response of 2dpf zebrafish embryos to sublethal dose (~80 CFU) of S. sonnei. Approximately 80% of WT GFP-S. sonnei injected embryos (and 100% of ΔO-Ag GFP-S. sonnei injected embryos) control infection (no detectable bacteria by fluorescence microscopy) by 48 hpi (A). Log10-transformed CFU counts from controller larvae (no detectable bacteria by fluorescence microscopy) infected in the HBV with GFP-ΔO-Ag (blue) or WT (red) S. sonnei. Prior to receiving the secondary lethal dose (~8000 CFU) of mCherry-S. sonnei, ~80% of WT GFP-S. sonnei injected controllers (and 100% of ΔO-Ag GFP-S. sonnei injected controllers) cleared the primary infection (no CFU detectable from plating). Experiments are cumulative of 4 (A) or 3 (B) biological replicates. Statistics: two-sided chi-square contingency test; ****p<0.0001. (PDF) [file ppat.1008006.s006.pdf]
