# Supplementary material for: Shigella sonnei infection of zebrafish reveals that O-antigen mediates neutrophil tolerance and dysentery incidence
Source: PLoS Pathog. 2019 Dec 12;15(12):e1008006. doi: 10.1371/journal.ppat.1008006 (PMC6980646; doi:10.1371/journal.ppat.1008006)

Figure S5. *S. sonnei* can resist phagolysosome acidification and promote neutrophil cell death in an O-antigen-dependent manner

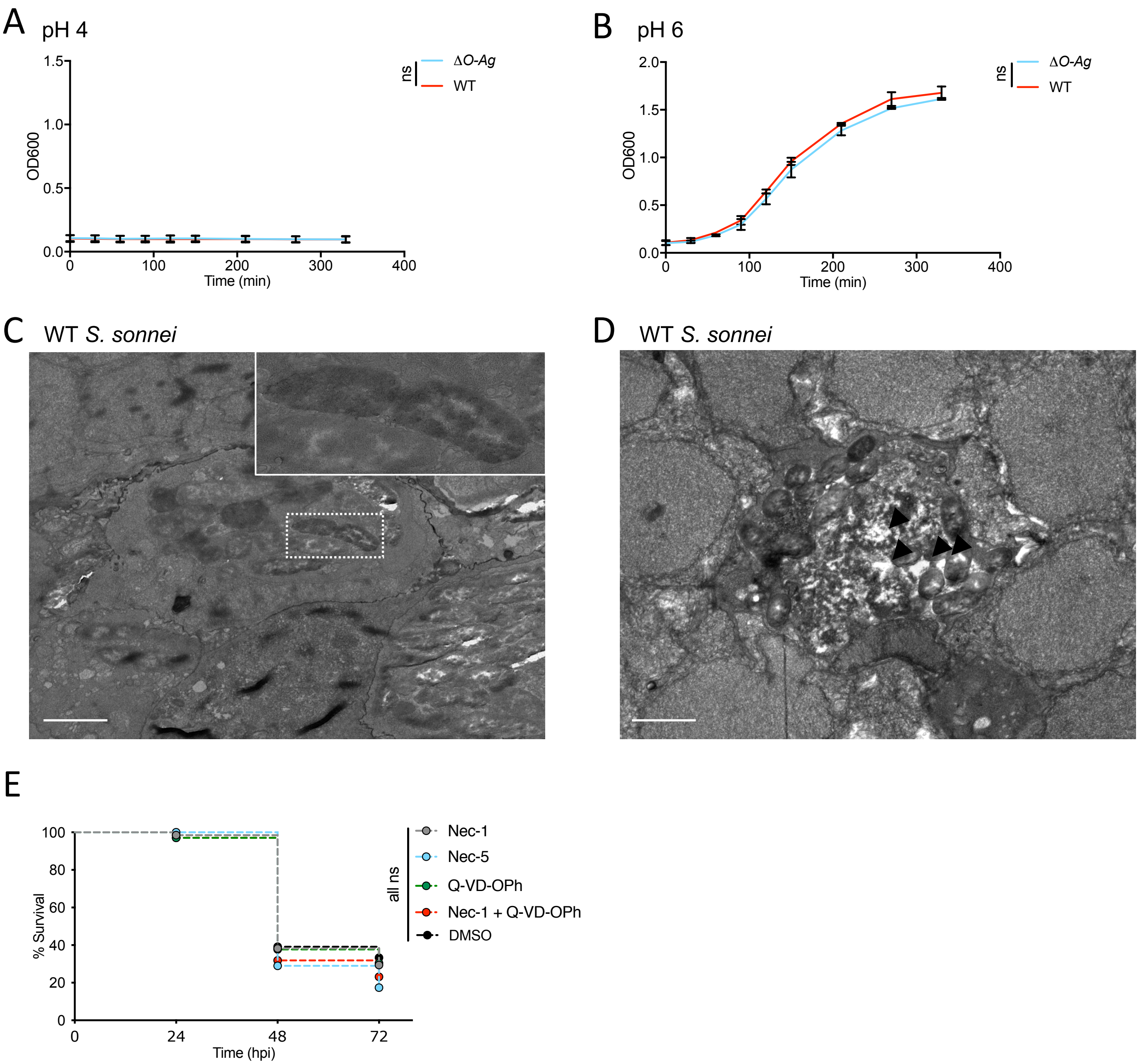

Supplement: S5 Fig — A,B. S. sonnei O-antigen contributes to acid tolerance in vitro. Growth curves of ΔO-Ag (blue) or WT (red) S. sonnei, cultured in tryptic soy broth adjusted to pH = 4 (A) or 6 (B). Statistics: unpaired t-test at the latest timepoint; ns, non-significant. C. S. sonnei can replicate within phagosomes. Transmission electron micrograph of an infected phagocyte from zebrafish larvae at 3 hpi with WT S. sonnei, showing a dividing S. sonnei cell. Scale bar = 4 μm. D. S. sonnei infection of zebrafish cell promotes morphological features of necrosis. Transmission electron micrograph of an infected neutrophil from a zebrafish larva at 3 hpi with WT S. sonnei, showing signs of necrotic cell death (arrowheads point at area of extranuclear chromatin degradation). Scale bar = 4 μm. E. Pharmacological inhibition of necroptosis and/or apoptosis/pyroptosis does not protect zebrafish larvae. Survival curves of larvae which were treated with Necrostatin-1 (grey), Necrostatin-5 (blue), Q-VD-OPh (green), Necrostatin-1 + Q-VD-OPh (red) or control DMSO vehicle (black) upon infection in the HBV with S. sonnei. Experiments are cumulative of 3 biological replicates. Bacterial input: ~7000 CFU. Statistics: Log-rank (Mantel-Cox) test; ns, non-significant. (PDF) [file ppat.1008006.s005.pdf]
