# Supplementary material for: Shigella sonnei infection of zebrafish reveals that O-antigen mediates neutrophil tolerance and dysentery incidence
Source: PLoS Pathog. 2019 Dec 12;15(12):e1008006. doi: 10.1371/journal.ppat.1008006 (PMC6980646; doi:10.1371/journal.ppat.1008006)
Supplement: S3 Table — (PDF) [file ppat.1008006.s009.pdf]

Table S3. Bacterial strains used in this study

Species	Strain background	Reporter construct	Mutation/ Complement	Notes
S. sonnei	53G (Formal *et al.,* 1966)	NA	WT	-
S. sonnei	53G	GFP from pFPV25.1 (Valdivia *et al.,* 2006)	WT	Carb^{R}
S. sonnei	53G	mCherry from pFPV-mcherry (Drecktrah *et al.,* 2008)	WT	Carb^{R}
S. sonnei	381 (Watson *et al.,* 2018)	GFP from pFPV25.1	WT	Clinical isolate H140860381 from C. Jenkins, Public Health England; Carb^{R}
S. sonnei	53G	GFP from pFPV25.1	*Δtssb* (This study)	T6SS mutant with *tssB* replaced with *aphA-3*, conferring Kanamycin resistance; Carb^{R}, Kan^{R}
S. sonnei	53G	GFP from pFPV25.1	*Δg4c* (Caboni *et al.,* 2015)	Capsule mutant with *g4c* cluster (genes *ymcDCBA, yccZ, etp*, *etk*) replaced with *erm* cassette conferring resistance to Erythromycin; Carb^{R}, Erm^{R}
S. sonnei	53G	GFP from pFPV25.1	*Δmxid* (Watson *et al.,* 2018)	T3SS mutant, with *mxiD* replaced with *aphA-3*, conferring Kanamycin resistance; Carb^{R}, Kan^{R}
S. sonnei	53G	GFP from pFPV25.1	-pSS This study, based on (McVicker *et al.,* 2016)	Phase II *S. sonnei*, spontaneously cured from pSS virulence plasmid; Carb^{R}
S. sonnei	53G	NA	*ΔO-Ag* (Watson *et al.,* 2019)	O-Antigen mutant, with O-Ag biosynthesis operon (*wbg*[illegible] *wzy,* IS630, *wbgVWXYZ*) replaced with *aphA-3,* c[illegible] Kanamycin resistance; Kan^{R}
S. sonnei	53G	GFP from pFPV25.1	*ΔO-Ag*	O-Antigen mutant with O-Ag biosynthesis operon (*wbgTU, wzx, wzy,* IS630, *wbgVWXYZ*) replaced with *aphA-3,* conferring kanamycin resistance; Carb^{R}, Kan^{R}
S. sonnei	53G	NA	*ΔO-Ag*$^{+pSSO\text{-}Ag}$ (Watson *et al.,* 2019)	*O-Ag* biosynthesis operon (*wzz, wbgTU, wzx, wzy,* IS630, *wbgVWXYZ* with 102 bp 5' of *wzz* and 76 bp 3' of *wbgZ*) from 53G inserted into pSEVA471:SmR expressed in a *S. sonnei* 53G *ΔO-Ag* background; Kan^{R}, Sm^{R}
S. flexneri	M90T-sm (Sansonetti *et al.,* 1982)	GFP (Mostowy *et al.,* 2010)	WT	Serotype 5a; Carb^{R}, naturally Sm^{R}
S. flexneri	2457T (Formal *et al.,* 1958)	GFP from pFPV25.1	WT	Serotype 2a; Carb^{R}

References

Formal SB, Kent TH, May HC, Palmer A, Falkow S, LaBrec EH. Protection of monkeys against experimental shigellosis with a living attenuated oral polyvalent dysentery vaccine. J Bacteriol. 1966 Jul;92(1):17-22.

Valdivia RH, Falkow S. Bacterial genetics by flow cytometry: rapid isolation of *Salmonella* typhimurium acid-inducible promoters by differential fluorescence induction. Mol Microbiol. 1996 Oct;22(2):367-78.

Drecktrah D, Levine-Wilkinson S, Dam T, Winfree S, Knodler LA, Schroer TA, Steele-Mortimer O. Dynamic behavior of *Salmonella*-induced membrane tubules in epithelial cells. Traffic. 2008 Dec;9(12):2117-29. doi:10.1111/j.1600-0854.2008.00830.x.

Caboni M, Pédron T, Rossi O, Goulding D, Pickard D, Citiulo F, MacLennan CA,Dougan G, Thomson NR, Saul A, Sansonetti PJ, Gerke C. An O antigen capsule modulates bacterial pathogenesis in *Shigella sonnei*. PLoS Pathog. 2015 Mar 20;11(3):e1004749. doi: 10.1371/journal.ppat.1004749.

Watson J, Jenkins C, Clements A. *Shigella sonnei* does not use amoebae as protective hosts. Appl Environ Microbiol. 2018 Apr 16;84(9). pii: e02679-17. doi:10.1128/AEM.02679-17.

McVicker G, Tang CM. Deletion of toxin-antitoxin systems in the evolution of *Shigella sonnei* as a host-adapted pathogen. Nat Microbiol. 2016 Nov 7;2:16204.doi:10.1038/nmicrobiol.2016.204.

Watson JL, Sanchez-Garrido J, Goddard PJ, Torraca V, Mostowy S, Shenoy AR, Clements A. *Shigella sonnei* O-antigen inhibits internalisation, vacuole escape and inflammasome activation. bioRxiv. 2019, 799379; doi:10.1101/799379.

Sansonetti PJ, Kopecko DJ, Formal SB. Involvement of a plasmid in the invasive ability of *Shigella flexneri*. Infect Immun. 1982 Mar;35(3):852-60.

Mostowy S, Bonazzi M, Hamon MA, Tham TN, Mallet A, Lelek M, Gouin E, Demangel C, Brosch R, Zimmer C, Sartori A, Kinoshita M, Lecuit M, Cossart P. Entrapment of intracytosolic bacteria by septin cage-like structures. Cell Host Microbe. 2010 Nov 18;8(5):433-44. doi:10.1016/j.chom.2010.10.009.

Formal SB, Dammin GJ, Labrec EH, Schneider H. Experimental *Shigella* infections: characteristics of a fatal infection produced in guinea pigs. J Bacteriol. 1958 May;75(5):604-10.
