# Supplementary material for: Shigella sonnei infection of zebrafish reveals that O-antigen mediates neutrophil tolerance and dysentery incidence
Source: PLoS Pathog. 2019 Dec 12;15(12):e1008006. doi: 10.1371/journal.ppat.1008006 (PMC6980646; doi:10.1371/journal.ppat.1008006)
Supplement: S4 Table — (PDF) [file ppat.1008006.s010.pdf]

Table S4. Primers and morpholinos used in this study

qPCR primers

Name	Geneid	Sequence (5'-3')	Ref.
eef1a1aFW	ENSDARG00000039502	AAGCTTGAAGACAACCCCAAGAGC	Herbst *et al.,* 2015
eef1a1aRV	ENSDARG00000039502	ACTCCTTTAATCACTCCCACCGCA	Herbst *et al.,* 2015
il1bFW	ENSDARG00000098700	GAACAGAATGAAGCACATCAAACC	Torraca *et al.,* 2017
il1bRV	ENSDARG00000098700	ACGGCACTGAATCCACCAC	Torraca *et al.,* 2017
cxcl18bFW	ENSDARG00000075045	TCTTCTGCTGCTGCTTGCGGT	Torraca *et al.,* 2017
cxcl18bRV	ENSDARG00000075045	GGTGTCCCTGCGAGCACGAT	Torraca *et al.,* 2017
cxcl8aFW	ENSDARG00000104795	TGTGTTATTGTTTTCCTGGCATTTC	Stockhammer *et al.,* 2009
cxcl8aRV	ENSDARG00000104795	GCGACAGCGTGGATCTACAG	Stockhammer *et al.,* 2009
mmp9FW	ENSDARG00000042816	CATTAAAGATGCCCTGATGTATCCC	Stockhammer *et al.,* 2009
mmp9RV	ENSDARG00000042816	AGTGGTGGTCCGTGGTTGAG	Stockhammer *et al.,* 2009

Morpholino oligonucleotides

Name	Geneid	Sequence (5'-3')	Notes	Ref.
ctrl Mo	-	CCTCTTACCTCAGTTACAATTTATA	Standard control morpholino	www.gene-tools.com
pu.1 (spi1b) Mo	ENSDARG00000000767	GATATACTGATACTCCATTGGTGGT	Splice blocking morpholino	Rhodes *et al.,* 2005

Primers used to create *S. sonnei* 53G *Δtssb* strain

Name	Sequence (5'-3')
#1	CTGGATAACCATACCCAAC
#2	GCAGCTCCAGCCTACACAGCTGCCTTCAAATTTTTTGC
#3	CTAAGGAGGATATTCATATGCCACCACTATTTCATTAAAC
#4	CATGCTGTCCACGGCCAG
#5	TGTGTAGGCTGGAGCTGCTTC
#6	CATATGAATATCCTCCTTAG
#7	GTTACTCCCTGTCTAACC
#8	GGTACGCCACCAGTGAAG

References

Herbst S, Shah A, Mazon Moya M, Marzola V, Jensen B, Reed A, Birrell MA, Saijo S, Mostowy S, Shaunak S, Armstrong-James D. Phagocytosis-dependent activation of a TLR9-BTK-calcineurin-NFAT pathway co-ordinates innate immunity to *Aspergillus fumigatus*. EMBO Mol Med. 2015 Mar;7(3):240-58. doi:10.15252/emmm.201404556.

Torraca V, Tulotta C, Snaar-Jagalska BE, Meijer AH. The chemokine receptor CXCR4 promotes granuloma formation by sustaining a mycobacteria-induced angiogenesis programme. Sci Rep. 2017 Mar 23;7:45061. doi:10.1038/srep45061.

Stockhammer OW, Zakrzewska A, Hegedûs Z, Spaink HP, Meijer AH. Transcriptome profiling and functional analyses of the zebrafish embryonic innate immune response to Salmonella infection. J Immunol. 2009 May 1;182(9):5641-53. doi:10.4049/jimmunol.0900082.

Rhodes J, Hagen A, Hsu K, Deng M, Liu TX, Look AT, Kanki JP. Interplay of pu.1 and gata1 determines myelo-erythroid progenitor cell fate in zebrafish. Dev Cell. 2005 Jan;8(1):97-108.
